# Supplementary material for: Non-pharmacological interventions for the reduction and maintenance of blood pressure in people with prehypertension: a systematic review protocol
Source: BMJ Open. 2024 Jan 22;14(1):e078189. doi: 10.1136/bmjopen-2023-078189 (PMC10806604; doi:10.1136/bmjopen-2023-078189)
Supplement: Supplementary data [file bmjopen-2023-078189supp001.pdf]

Non-Pharmacological intervention for PHT systematic review

Medline Search Strategy

Search conducted May 26th 2023

Search Number	Query	Hits
1	Prehypertension/	1144
2	prehypertensi*.ti,ab,kw,kf.	2946
3	pre-hypertensi*.ti,ab,kw,kf.	925
4	(pre-htn or prehtn).tw.	113
5	((stage 1 or stage one) adj hypertensi*).tw.	666
6	(elevated blood pressure or elevated bp).ti.	866
7	high-normal blood pressure.tw.	409
8	raised blood pressure.ti.	83
9	borderline hypertensi*.tw.	1403
10	early hypertensi*.tw.	365
11	raised systolic blood pressure.tw.	75
12	raised diastolic blood pressure.tw.	16
13	("at risk" adj1 hypertensi*).tw.	43
14	((("120" adj2 "129") or ("130" adj2 "139") or ("120" adj2 "139") or ("80" adj2 "89")) and (blood pressure or bp)).tw.	964
15	or/1-14	7975
16	exp randomized controlled trial/	594769
17	controlled clinical trial.pt.	95314
18	randomi#ed.ab.	721331
19	placebo.ab.	238387
20	randomly.ab.	408822
21	trial.ab.	649200
22	groups.ab.	2520111
23	or/16-22	3638767
24	exp animals/ not humans.sh.	5123796
25	23 not 24	3135655
26	15 and 25	2372
