# Supplementary material for: Non-pharmacological interventions for the reduction and maintenance of blood pressure in people with prehypertension: a systematic review protocol
Source: BMJ Open. 2024 Jan 22;14(1):e078189. doi: 10.1136/bmjopen-2023-078189 (PMC10806604; doi:10.1136/bmjopen-2023-078189)
Supplement: Author's manuscript [file bmjopen-2023-078189.draft_revisions.pdf]

BMJ Open is committed to open peer review. As part of this commitment we make the peer review history of every article we publish publicly available.

When an article is published we post the peer reviewers' comments and the authors' responses online. We also post the versions of the paper that were used during peer review. These are the versions that the peer review comments apply to.

The versions of the paper that follow are the versions that were submitted during the peer review process. They are not the versions of record or the final published versions. They should not be cited or distributed as the published version of this manuscript.

BMJ Open is an open access journal and the full, final, typeset and author-corrected version of record of the manuscript is available on our site with no access controls, subscription charges or pay-per-view fees (http://bmjopen.bmj.com).

If you have any questions on BMJ Open's open peer review process please email info.bmjopen@bmj.com

BMJ Open

Non-pharmacological interventions for the reduction and maintenance of blood pressure in people with prehypertension: A systematic review protocol

Journal:	*BMJ Open*
Manuscript ID	bmjopen-2023-078189
Article Type:	Protocol
Date Submitted by the Author:	26-Jul-2023

Complete List of Authors:	Bray, Emma; University of Central Lancashire, Senior Research Fellow, Stroke Research Team, School of Nursing<br>Georgiou, Rachel; University of Central Lancashire, Senior Research Fellow, Stroke Research Team, School of Nursing<br>Hives, Lucy; University of Central Lancashire, Research Associate, School of Community Health and Midwifery<br>Iqbal, Nafisa; University of Central Lancashire, Stroke Team<br>Benedetto, Valerio; University of Central Lancashire, Research Fellow, Synthesis, Economic Evaluation and Decision Science Group<br>Spencer, Joseph; University of Central Lancashire, Senior Research Assistant, Research Facilitation and Delivery Unit<br>Harris, Cath; University of Central Lancashire, Information Specialist, Synthesis, Economic Evaluation and Decision Science Group<br>Clegg, Andrew; University of Central Lancashire, Professor of Health Services Research, Synthesis, Economic Evaluation and Decision Science Group<br>Williams, Nefyn; University of Liverpool, Professor of Primary Care, Department of Primary Care and Mental Health<br>Rutter, Paul; University of Portsmouth, Professor of Pharmacy Practice, School of Biomedical Sciences<br>Watkins, Caroline; University of Central Lancashire, Professor of Stroke and Older People’s Care, Stroke Research Team

Keywords:	Blood Pressure, Prehypertension, Primary Prevention, Systematic Review

SCHOLARONE™
Manuscripts

Non-pharmacological interventions for the reduction and maintenance of blood pressure in people with prehypertension: A systematic review protocol

CORRESPONDING AUTHOR: Dr Emma Bray, EBray@uclan.ac.uk

Authors

Dr Emma Bray[1], Rachel Georgiou[1], Lucy Hives[2], Nafisa Iqbal[1], Dr Valerio Benedetto[3], Joseph Spencer[4], Catherine Harris[3], Prof Andrew Clegg[3], Prof Nefyn Williams[5], Prof Paul Rutter[6], Prof Caroline Watkins

[1] Stroke Research Team, School of Nursing, Faculty of Health and Care, University of Central Lancashire, PR1 2HE, UK.
[2] School of Community Health and Midwifery, University of Central Lancashire, UK.
[3] Synthesis, Economic Evaluation and Decision Science Group; Research Fellow, Health Technology Assessment Unit, University of Central Lancashire, UK.
[4] Research Facilitation and Delivery Unit, University of Central Lancashire, UK.
[5] Department of Primary Care and Mental Health, Institute of Population Health, University of Liverpool, UK.
[6] School of Biomedical Sciences, University of Portsmouth, Portsmouth, UK.

Word count: 2092

ABSTRACT

Introduction

Prehypertension is defined as blood pressure that is above the normal range but not high enough to be classed as hypertension. Prehypertension is a warning of development of hypertension as well as a risk for cardiovascular disease, heart attack and stroke. In the UK, non-pharmacological interventions are recommended for prehypertension management, but no reviews have focused on the effectiveness of these types of interventions solely in people with prehypertension. Therefore, the proposed systematic review will assess the clinical- and cost–effectiveness of non-pharmacological interventions in reducing or maintaining blood pressure in pre-hypertensive people.

Methods and analysis

This systematic review will follow the Preferred Reporting Items for Systematic Reviews and Meta-Analyses guidelines. The databases/ trial registries that will be searched to identify relevant randomised controlled trials (RCTs) and economic evaluations include Medline, EMBASE, CINAHL, PsycINFO, CENTRAL, the World Health Organization International Clinical Trials Registry Platform, ClinicalTrials.gov, Cochrane Library, Scopus, and the International HTA Database. Search terms have been identified by the team including an information specialist. Three reviewers will be involved in the study selection process. Risk of bias will be evaluated using the Cochrane risk-of-bias tool for RCTs, and the Consensus Health Economic Criteria list for economic evaluations. Findings from the included studies will be tabulated and synthesised narratively. Heterogeneity will be assessed through visual inspection of forest plots and the calculation of the Chi^2 and I^2 statistics, and causes of

For peer review only - http://bmjopen.bmj.com/site/about/guidelines.xhtml

heterogeneity will be assessed where sufficient data are available. If possible, we plan to investigate differential effects on specific sub-groups and from different types of interventions using meta-regression.

Ethics and dissemination

Ethical approval is not needed. Results will be published in a peer-reviewed journal, disseminated via the wider study website and shared with the study sites and participants.

Registration details: The review is registered with Prospero (CRD420232433047)

KEYWORDS: Prehypertension; blood pressure; non-pharmacological interventions

Strengths and limitations of this study

- To our knowledge, the systematic review will be the first to provide an up-to-date synthesis on non-pharmacological interventions to reduce blood pressure in individuals in the prehypertensive range.
- A comprehensive search strategy has been designed collaboratively with a skilled information specialist.
- There will be no restrictions applied to the searches regarding language or date of publication.
- This review will only include randomised controlled trials and comparative economic evaluations. Other study designs will be excluded.

For peer review only

INTRODUCTION

People with blood pressure (BP) in the prehypertension (PHT) range (120-139/80-89 mmHg), are at increased risk, compared to those with normal BP, of developing hypertension [1,2] and other cardiovascular disease (CVD)-related conditions, independent of progression to hypertension.[3-9] The estimated number of people with PHT is substantial, with around 40% of adults attending primary care clinics falling into this category.[10] BP exists on a continuum following a normal distribution, with risk of CVD increasing as BP increases. PHT, also known as “high-normal”, “elevated” or “raised” BP, lies between normal BP (<120/80 mmHg) and hypertension (>=140/90 mmHg).

PHT itself however is not regarded as an illness, rather it is a warning of “an insidious progression” of BP to problematic levels.[11] It is starting to be recognised that prevention needs to be at the heart of future healthcare[12] with a change of emphasis from fixing ill-health to protecting good health. PHT can be a useful sign to identify and alert those at-risk of developing hypertension and CVD, so they can take action to prevent, delay or reduce progression to disease status.[11]

In the UK, PHT does not require drug intervention as in hypertension. Rather, guidance [13] recommends that PHT management should be focused on lifestyle modification, and research has demonstrated that lifestyle modifications at the “pre-risk” stage could provide lifetime benefits; for example, by making lifestyle changes, people with PHT can significantly reduce both their risk of developing hypertension [14] and also reverse PHT to normotensive levels.[15,16] A review of current evidence on effective non-pharmacological interventions for PHT would help guide research and practice in this area. To our knowledge, the only review[17] on this topic included studies of both PHT

For peer review only - http://bmjopen.bmj.com/site/about/guidelines.xhtml

and hypertensive participants making it difficult to determine what might be effective for those specifically with PHT. The aim of this review is to assess the clinical and cost-effectiveness of different non-pharmacological interventions for reducing BP and maintaining BP in adults with PHT.

Review question

What is the clinical- and cost-effectiveness of non-pharmacological interventions for reducing and/or maintaining blood pressure in adults with prehypertension, compared to usual care, or other non-pharmacological interventions?

METHODS AND ANALYSIS

Study Design

This systematic review will be conducted and reported following the Preferred Reporting Items for Systematic Reviews and Meta-Analyses (PRISMA) guidelines.

Search Strategy

Separate searches to identify randomised controlled trials (RCTs) or economic evaluations will be conducted. Seven databases and trial registries will be searched to identify relevant RCTs: Medline, EMBASE, CINAHL, PsycINFO, Cochrane Central Register of Controlled Trials (CENTRAL), the World Health Organization International Clinical Trials Registry Platform, and ClinicalTrials.gov. Medline, EMBASE, CINAHL, PsycINFO, Cochrane Library via Wiley (all databases), Scopus, and the International HTA Database will be searched to identify economic evaluations. We will screen the reference lists of all included studies and relevant review articles for any additional studies. There will be no restrictions applied to the searches regarding language or date of publication.

The search terms were identified by the team (including an information specialist) via an iterative process. The search strategies include keywords and subject headings relating to prehypertension which were combined with the Cochrane Highly Sensitive Search Strategy[18] for identifying Randomized Trials in Medline (sensitivity-maximising version) and the NHS EED Search Filter[19] to identify economic evaluations. The search strategies will be adapted for use in each database and combined with database-specific search filters for RCTs and economic evaluations where these are available. The search strategies for Medline can be found in Appendix 1.

Study Selection

Search results will be imported into EndNote (version X9, Clarivate Analytics, Philadelphia, PA), where duplicates will be removed. The final list for screening will then be imported into Rayyan®. Screening for the studies of clinical effectiveness and cost-effectiveness will be done as two separate processes. One reviewer will screen all the titles and abstracts of the search results to identify relevant articles, and a second reviewer will review at least 10% to ensure consistency. A third reviewer will then check those studies deemed as potentially eligible for the next stage. One reviewer will then independently screen the full texts of the potentially eligible articles, and a second reviewer will check the ones selected for inclusion, to determine final inclusion in the review. Disagreements will be resolved via discussion, and where necessary through the involvement of a third reviewer. The study selection process will be presented using a PRISMA flow diagram.

For peer review only - http://bmjopen.bmj.com/site/about/guidelines.xhtml
For peer review only

Inclusion Criteria

Only RCTs and comparative economic evaluations (including costs and/or consequences) will be eligible for inclusion in the review.

Condition or domain being studied

Prehypertension (PHT). Studies will also be included where samples contain people both with PHT AND hypertension or normotension if the data for the PHT group is presented and can be extracted separately (e.g., within the text or data tables).

Participants/population

Inclusion criteria: People aged 18 years old or older with blood pressure in the prehypertension range defined as systolic blood pressure 120-139mmHg and/or diastolic blood pressure 80-89 mmHg.

Exclusion criteria: We will exclude studies where participants have/had a diagnosis of hypertension, are on anti-hypertensive medication, are pregnant, have a life limiting disease or have previously had a cardiovascular event (e.g., heart attack, stroke, transient ischaemic attack) or have a pre-existing cardiovascular disease.

Intervention(s), exposure(s)

All non-pharmacological interventions will be considered, regardless of presentation or delivery mode (e.g., face-to-face, group, one-to-one, self-directed, etc), for inclusion. This will include investigations of foods and/ or compounds found in foods (e.g., caffeine, flavonoids).

Comparator(s)/control

Studies will be included if they compare non-pharmacological interventions to usual care, or if they compare different types of non-pharmacological interventions to each other.

Context

Study setting may be primary or secondary care, or community settings.

Main outcome(s)

Studies reporting a change in BP (both systolic and diastolic) between intervention and comparator will be included. BP could be office, ambulatory or self-monitored readings. We will also include studies that report a change in cardiovascular morbidity (cardiovascular disease, hypertension, myocardial infarction, or stroke/transient ischaemic attack), and those that include a change in costs, quality of life (QoL) and cost-effectiveness as outcomes. All follow-up lengths will be included, with the differential effects associated with different durations explored in sub-group analyses.

For peer review only

For peer review only - http://bmjopen.bmj.com/site/about/guidelines.xhtml

Measures of effect

The measures of effect for change in BP and change in QoL will be the mean difference, standard deviation, and 95% confidence interval (CI).

For occurrence of CVD, hypertension, stroke, TIA, and MI, the measure of effect will be the odds ratio (OR).

For cost-effectiveness, we will consider mean differences in costs and QoL between comparators (and any dispersion measures as reported by the included studies), as well as cost per marginal gain in the specific QoL measures employed by the studies (e.g., quality-adjusted life years or QALYs).

Data extraction (selection and coding)

Two reviewers will independently extract data from the agreed eligible studies. A coding scheme will be developed and piloted to ensure all relevant data is captured and to ensure both reviewers are extracting data in a standardised manner. It is envisaged the following data will be extracted:

- *Study context:* authors, study title, journal, year of publication, location of study, funding details, conflicts of interest;
- *Design and Methods:* method of randomisation, details of study sites (number, location, setting etc), variables of interest, data collection tools, planned follow-up timings, key attributes of economic evaluations (e.g., perspective, design, time horizon, characteristics of cost and outcome data, cost-effectiveness measure employed);
- *Participants:* number, basic demographics (age, gender, ethnicity), blood pressure, co-morbidities;
- *Interventions:* type of intervention, details of the intervention including length, frequency and duration, comparison, mode of delivery, provider, content and components;
- *Outcomes:* systolic blood pressure, diastolic blood pressure, presence of cardiovascular morbidity (hypertension, MI, stroke, TIA, cardiovascular disease), costs, QoL and cost-effectiveness at baseline and the stated follow-up time points of each study.

Risk of bias (quality) assessment

Two reviewers will independently critically appraise the eligible studies. Risk of bias will be evaluated using the Cochrane risk-of-bias tool (RoB) for RCTs, and the Consensus Health Economic Criteria (CHEC) list for economic evaluations[20].

Strategy for data synthesis

Studies will be synthesised through narrative reviews with tabulation of results of included studies. Where possible, treatment effects for all comparisons and outcomes will be synthesised through meta-analyses, with the approach taken dependant on the outcome assessed and the data available. Dichotomous data will be presented as risk ratios with 95% CIs. Continuous data will be synthesised as weighted mean differences (MD) when outcomes are assessed on the same scale, or standardised weighted mean difference (SMD) when different scales are used to measure the same underlying construct, with 95% CI. Where the outcomes represent time-to-event data (e.g., overall survival), the

For peer review only

(log) hazard ratio with 95% CI will be used as the summary measure. Heterogeneity will be assessed through visual inspection of forest plots and the calculation of the Chi^2 and I^2 statistics. Causes of heterogeneity will be assessed where sufficient data is available, including factors such as participant characteristics (e.g., age, sex), baseline BP, method of obtaining BP readings (office, ambulatory, home), duration of intervention, co-morbidities and delivery/ mode of intervention. Where appropriate, these will be investigated further through sub-group analyses and through meta-regression. Sensitivity analyses will explore possible causes of methodological heterogeneity, where sufficient data are available. This would include assessing the effects of studies that may be affected by factors such as risk of bias associated with allocation concealment, high loss to follow-up or lack of blinding in assessment of outcomes. Where data are missing, particularly measures of variation (e.g., standard deviation), we will contact study authors or impute values. It is likely that the analysis will focus on direct comparisons of intervention effects through pairwise meta-analyses. Where evidence allows, we will consider conducting network meta-analysis through both direct and indirect evidence within connected networks of trials. Pairwise meta-analyses of direct comparisons will be conducted using STATA v17 (version 17, StataCorp, Texas, USA) or Comprehensive Meta-analysis v4, while NMAs will be estimated using the WinBUGS software (version 1.4.3) (MRC Biostatistics Unit, Cambridge, UK)(http://www.mrc-bsu.cam.ac.uk/bugs/winbugs/contents.shtml).

Analysis of subgroups or subsets

Depending on the variety of non-pharmacological interventions and studies' populations and on the available data, we plan to investigate differential effects on specific sub-groups (e.g., by age, sex, BP levels, co-morbidities, health-related quality of life), and from different types of interventions (e.g., by duration, setting, mode of delivery) using meta-regression. Where this quantitative approach is not possible, we will discuss the sub-group effects narratively.

Assessment of certainty of evidence

Where relevant, we will assess the level of certainty of the evidence found in the included studies using the Grading of Recommendations, Assessment, Development, and Evaluations (GRADE) framework[21].

PATIENT AND PUBLIC INVOLVEMENT

Patients and the public were not directly involved in designing this systematic review protocol. The systematic review is connected to a larger programme of work called REVERSE (Risk Education InterVEntion for Raised blood preSsurE). REVERSE aims to investigate the feasibility of blood pressure self-monitoring for people with prehypertension, which was co-produced with members of the public with prehypertension and hypertension. The results of both the systematic review of non-pharmacological interventions and feasibility study of self-monitoring will help with co-producing future lifestyle interventions, with the aim of preventing hypertension and associated conditions, which will be tested in future effectiveness studies.

ETHICS AND DISSEMINATION

Ethical approval is not needed for this systematic review. The review is registered with Prospero (CRD420232433047), and any important protocol amendments will be updated on the record. It is intended to publish the completed review in a peer-reviewed journal. Results will also be

For peer review only - http://bmjopen.bmj.com/site/about/guidelines.xhtml

disseminated via the REVERSE study website and will be shared with the REVERSE study sites and participants.

TWITTER

@ReverseStudy

WEBSITE

www.reversestudy.co.uk

AUTHORS CONTRIBUTIONS

EB was responsible for the conception, design and writing of the manuscript. LH, JS, EB, RG, CW, VB, NW, PR and AC contributed to the conception and design. EB, LH, VB and NI contributed to the writing of the manuscript. CH designed the search strategy and performed the preliminary database searches. All authors read and approved the final manuscript. EB is the guarantor of the review.

FUNDING STATEMENT

EB holds a NIHR RfPB grant (NIHR201028). This work is related to this funding but is not directly funded by it. The views expressed are those of the author(s) and not necessarily those of the NIHR or the Department of Health and Social Care.

COMPETING INTERESTS STATEMENT

VB, CH, AC , JS and CW are part funded by the National Institute for Health Research Applied Research Collaboration North West Coast (NIHR ARC NWC). The views expressed are those of the authors and not necessarily those of the NHS, the NIHR, or the Department of Health and Social Care.

For peer review only - http://bmjopen.bmj.com/site/about/guidelines.xhtml

REFERENCES

[1] Wang Q, Wang Z, Yao W, Wu X, Huang J, Huang L, et al. Anthropometric indices predict the development of hypertension in normotensive and pre- hypertensive middle-aged women in Tianjin, China: A prospective cohort study. *Medical Science Monitor.* 2018;24:1871-9.

[2] Zheng L, Sun Z, Zhang X, Xu C, Li J, Hu D, et al. Predictors of progression from prehypertension to hypertension among rural Chinese adults: Results from Liaoning Province. *European Journal of Cardiovascular Prevention and Rehabilitation*. 2010;17(2):217-22.

[3] Huang Y, Su L, Cai X, Mai W, Wang S, Hu Y, et al. Association of all-cause and cardiovascular mortality with prehypertension: A meta-analysis. *American Heart Journal*. 2014;167(2):160-8.e1.

[4] Guo X, Yingxian S. Quantitative association between prehypertension and different types of cardiovascular outcomes and diabetes: A systematic review and meta-analysis of prospective studies worldwide. *Heart*. 2013;3:A115.

[5] Li Y, Xia P, Xu L, Wang Y, Chen L. A Meta-analysis on prehypertension and chronic kidney disease. *PLoS ONE*. 2016;11(6):e0156575.

[6] Kanno A, Kikuya M, Ohkubo T, Hashimoto T, Satoh M, Hirose T, et al. Pre-hypertension as a significant predictor of chronic kidney disease in a general population: The Ohasama Study. *Nephrology Dialysis Transplantation*. 2012;27(8):3218-23.

[7] Huang Y, Cai X, Liu C, Zhu D, Hua J, Hu Y, et al. Prehypertension and the risk of coronary heart disease in Asian and western populations: A meta-analysis. *Journal of the American Heart Association*. 2015;4(2):e001519.

[8] Lee M, Saver JL, Chang B, Chang KH, Lin K, Ovbiagele B. Baseline pre-hypertension and risk of incident stroke: A meta-analysis. *Stroke*. 2011;42(3):e49-e50.

[9] Winegarden CR. From "prehypertension" to hypertension? Additional evidence. *Annals of Epidemiology*. 2005;15(9):720-5.

[10] Joffres M, Falaschetti E, Gillespie C, Robitaille C, Loustalot F, Poulter N, et al. Hypertension prevalence, awareness, treatment and control in national surveys from England, the USA and Canada, and correlation with stroke and ischaemic heart disease mortality: a cross-sectional study. *BMJ Open*. 2013;3:e003423.

[11] Collier SR, Landram MJ. Treatment of prehypertension: lifestyle and/or medication. *Vascular Health and Risk Management.* 2012;8:613-19.

[12] National Health Service. *The NHS Long Term Plan*. Available at: https://www.longtermplan.nhs.uk/publication/nhs-long-term-plan/ [Accessed 19th February 2019].

[13] Whelton PK, Carey RM, Aronow WS, Casey DE Jr, Collins KJ, Dennison Himmelfarb C, et al. 2017 ACC/AHA/AAPA/ABC/ACPM/AGS/APhA/ASH/ASPC/NMA/PCNA guideline for the prevention, detection, evaluation, and management of high blood pressure in adults: executive summary: a report of the American College of Cardiology/American Heart Association Task Force on Clinical Practice Guidelines. *Hypertension*. 2018;71:e13-e115.

[14] Chiu YH, Wu SC, Tseng CD, Yen MF, Chen TH. Progression of pre-hypertension, stage 1 and 2 hypertension (JNC 7): a population-based study in Keelung, Taiwan (Keelung Community-based Integrated Screening No. 9). *Journal of Hypertension*. 2006;24(5):821-8.

[15] Moore LL, Visioni AJ, Qureshi MM, Bradlee ML, Ellison RC, D-Agostino R. Weight loss in overweight adults and the long-term risk of hypertension: The Framingham Study. *Archives of Internal Medicine*. 2005;165:1298–303.

[16] Padilla J, Wallace JP, Park S. Accumulation of physical activity reduces blood pressure in pre- and hypertension. *Medicine and Science in Sports and Exercise*. 2005;37:1264–75.

For peer review only

For peer review only - http://bmjopen.bmj.com/site/about/guidelines.xhtml

[17] Fu J, Liu Y, Zhang L, Zhou L, Li D, Quan H, et al. Nonpharmacologic Interventions for Reducing Blood Pressure in Adults With Prehypertension to Established Hypertension. *Journal of the American Heart Association.* 2020;9(19):e016804.

[18] Lefebvre C, Glanville J, Briscoe S, Featherstone R, Littlewood A, Marshall C, et al. Chapter 4: Searching for and selecting studies. In: Higgins JPT, Thomas J, Chandler J, Cumpston M, Li T, Page MJ, Welch VA (editors). *Cochrane Handbook for Systematic Reviews of Interventions* version 6.3 (updated February 2022). Cochrane, 2022. Available: www.training.cochrane.org/handbook.

[19] University of York Centre for Reviews and Dissemination. How are studies identified for NHS EED. 2009. Available: https://www.crd.york.ac.uk/crdweb/searchstrategies.asp [Accessed 15 May 2023].

[20] Evers S, Goossens M, De Vet H, Van Tulder M, Ament A. Criteria list for assessment of methodological quality of economic evaluations: Consensus on Health Economic Criteria. *International Journal of Technology Assessment in Health Care*. 2005;21(2):240-245.

[21] Schünemann HJ, Higgins JPT, Vist GE, Glasziou P, Akl EA, Skoetz N, Guyatt GH; on behalf of the Cochrane GRADEing Methods Group and the Cochrane Statistical Methods Group. Cochrane Handbook for Systematic Reviews of Interventions version 6.3, Section 14.3. 2022. Available: https://training.cochrane.org/handbook/current/chapter-14#section-14-3.

For peer review only

For peer review only - http://bmjopen.bmj.com/site/about/guidelines.xhtml

Non-Pharmacological intervention for PHT systematic review

Medline Search Strategy

Search conducted May 26th 2023

Search Number	Query	Hits
1	Prehypertension/	1144
2	prehypertensi*.ti,ab,kw,kf.	2946
3	pre-hypertensi*.ti,ab,kw,kf.	925
4	(pre-htn or prehtn).tw.	113
5	((stage 1 or stage one) adj hypertensi*).tw.	666
6	(elevated blood pressure or elevated bp).ti.	866
7	high-normal blood pressure.tw.	409
8	raised blood pressure.ti.	83
9	borderline hypertensi*.tw.	1403
10	early hypertensi*.tw.	365
11	raised systolic blood pressure.tw.	75
12	raised diastolic blood pressure.tw.	16
13	("at risk" adj1 hypertensi*).tw.	43
14	((("120" adj2 "129") or ("130" adj2 "139") or ("120" adj2 "139") or ("80" adj2 "89")) and (blood pressure or bp)).tw.	964
15	or/1-14	7975
16	exp randomized controlled trial/	594769
17	controlled clinical trial.pt.	95314
18	randomi#ed.ab.	721331
19	placebo.ab.	238387
20	randomly.ab.	408822
21	trial.ab.	649200
22	groups.ab.	2520111
23	or/16-22	3638767
24	exp animals/ not humans.sh.	5123796
25	23 not 24	3135655
26	15 and 25	2372

For peer review only

Reporting checklist for protocol of a systematic review and meta analysis.

Based on the PRISMA-P guidelines.

Instructions to authors

Complete this checklist by entering the page numbers from your manuscript where readers will find each of the items listed below.

Your article may not currently address all the items on the checklist. Please modify your text to include the missing information. If you are certain that an item does not apply, please write "n/a" and provide a short explanation.

Upload your completed checklist as an extra file when you submit to a journal.

In your methods section, say that you used the PRISMA-Preporting guidelines, and cite them as:

Moher D, Shamseer L, Clarke M, Ghersi D, Liberati A, Petticrew M, Shekelle P, Stewart LA. Preferred Reporting Items for Systematic Review and Meta-Analysis Protocols (PRISMA-P) 2015 statement. Syst Rev. 2015;4(1):1.

		Reporting Item	Page Number
**Title**			
Identification	#1a	Identify the report as a protocol of a systematic review	1
Update	#1b	If the protocol is for an update of a previous systematic review, identify as such	n/a
**Registration**			
	#2	If registered, provide the name of the registry (such as 7PROSPERO) and registration number	2 & 7
**Authors**			
Contact	#3a	Provide name, institutional affiliation, e-mail address of all protocol authors; provide physical mailing address of corresponding author	1
Contribution	#3b	Describe contributions of protocol authors and identify the guarantor of the review	7

For peer review only

**Amendments**			
	#4	If the protocol represents an amendment of a previously completed or published protocol, identify as such and list changes; otherwise, state plan for documenting important protocol amendments	7
**Support**			
Sources	#5a	Indicate sources of financial or other support for the review	8
Sponsor	#5b	Provide name for the review funder and / or sponsor	8
Role of sponsor or funder	#5c	Describe roles of funder(s), sponsor(s), and / or institution(s), if any, in developing the protocol	8
**Introduction**			
Rationale	#6	Describe the rationale for the review in the context of what is already known	3
Objectives	#7	Provide an explicit statement of the question(s) the review will address with reference to participants, interventions, comparators, and outcomes (PICO)	3
**Methods**			
Eligibility criteria	#8	Specify the study characteristics (such as PICO, study design, setting, time frame) and report characteristics (such as years considered, language, publication status) to be used as criteria for eligibility for the review	4-5
Information sources	#9	Describe all intended information sources (such as electronic databases, contact with study authors, trial registers or other grey literature sources) with planned dates of coverage	4
Search strategy	#10	Present draft of search strategy to be used for at least one electronic database, including planned limits, such that it could be repeated	Appendix 1
Study records - data management	#11a	Describe the mechanism(s) that will be used to manage records and data throughout the review	4
Study records - selection process	#11b	State the process that will be used for selecting studies (such as two independent reviewers) through each phase of the review (that is, screening, eligibility and inclusion in meta-analysis)	4
Study records - data	#11c	Describe planned method of extracting data from reports (such as	5-6

For peer review only

collection process		piloting forms, done independently, in duplicate), any processes for obtaining and confirming data from investigators	
Data items	#12	List and define all variables for which data will be sought (such as PICO items, funding sources), any pre-planned data assumptions and simplifications	6
Outcomes and prioritization	#13	List and define all outcomes for which data will be sought, including prioritization of main and additional outcomes, with rationale	6
Risk of bias in individual studies	#14	Describe anticipated methods for assessing risk of bias of individual studies, including whether this will be done at the outcome or study level, or both; state how this information will be used in data synthesis	6
Data synthesis	#15a	Describe criteria under which study data will be quantitatively synthesised	6-7
Data synthesis	#15b	If data are appropriate for quantitative synthesis, describe planned summary measures, methods of handling data and methods of combining data from studies, including any planned exploration of consistency (such as I2, Kendall's τ)	6-7
Data synthesis	#15c	Describe any proposed additional analyses (such as sensitivity or subgroup analyses, meta-regression)	6-7
Data synthesis	#15d	If quantitative synthesis is not appropriate, describe the type of summary planned	n/a
Meta-bias(es)	#16	Specify any planned assessment of meta-bias(es) (such as publication bias across studies, selective reporting within studies)	6
Confidence in cumulative evidence	#17	Describe how the strength of the body of evidence will be assessed (such as GRADE)	6

None The PRISMA-P elaboration and explanation paper is distributed under the terms of the Creative Commons Attribution License CC-BY. This checklist can be completed online using https://www.goodreports.org/, a tool made by the EQUATOR Network in collaboration with Penelope.ai

For peer review only

BMJ Open

Non-pharmacological interventions for the reduction and maintenance of blood pressure in people with prehypertension: A systematic review protocol

Journal:	*BMJ Open*
Manuscript ID	bmjopen-2023-078189.R1
Article Type:	Protocol
Date Submitted by the Author:	06-Oct-2023
Complete List of Authors:	Bray, Emma; University of Central Lancashire, Senior Research Fellow, Stroke Research Team, School of Nursing<br>Georgiou, Rachel; University of Central Lancashire, Senior Research Fellow, Stroke Research Team, School of Nursing<br>Hives, Lucy; University of Central Lancashire, Research Associate, School of Community Health and Midwifery<br>Iqbal, Nafisa; University of Central Lancashire, Stroke Team<br>Benedetto, Valerio; University of Central Lancashire, Research Fellow, Synthesis, Economic Evaluation and Decision Science Group<br>Spencer, Joseph; University of Central Lancashire, Senior Research Assistant, Research Facilitation and Delivery Unit<br>Harris, Cath; University of Central Lancashire, Information Specialist, Synthesis, Economic Evaluation and Decision Science Group<br>Clegg, Andrew; University of Central Lancashire, Professor of Health Services Research, Synthesis, Economic Evaluation and Decision Science Group<br>Williams, Nefyn; University of Liverpool, Professor of Primary Care, Department of Primary Care and Mental Health<br>Rutter, Paul; University of Portsmouth, Professor of Pharmacy Practice, School of Biomedical Sciences<br>Watkins, Caroline; University of Central Lancashire, Professor of Stroke and Older People's Care, Stroke Research Team
**Primary Subject Heading**:	Public health
Secondary Subject Heading:	Cardiovascular medicine, Health economics
Keywords:	Blood Pressure, Prehypertension, Primary Prevention, Systematic Review

SCHOLARONE™
Manuscripts

Non-pharmacological interventions for the reduction and maintenance of blood pressure in people with prehypertension: A systematic review protocol

CORRESPONDING AUTHOR: Dr Emma Bray, EBray@uclan.ac.uk

Authors

Dr Emma Bray[1], Rachel Georgiou[1], Lucy Hives[2], Nafisa Iqbal[1], Dr Valerio Benedetto[3], Joseph Spencer[4], Catherine Harris[3], Prof Andrew Clegg[3], Prof Nefyn Williams[5], Prof Paul Rutter[6], Prof Caroline Watkins[1]

1. Stroke Research Team, School of Nursing, Faculty of Health and Care, University of Central Lancashire, PR1 2HE, UK.
2. School of Community Health and Midwifery, University of Central Lancashire, UK.
3. Synthesis, Economic Evaluation and Decision Science Group; Research Fellow, Health Technology Assessment Unit, University of Central Lancashire, UK.
4. Research Facilitation and Delivery Unit, University of Central Lancashire, UK.
5. Department of Primary Care and Mental Health, Institute of Population Health, University of Liverpool, UK.
6. School of Biomedical Sciences, University of Portsmouth, Portsmouth, UK.

For peer review only

Word count: 2277

ABSTRACT

Introduction

Prehypertension is defined as blood pressure that is above the normal range but not high enough to be classed as hypertension. Prehypertension is a warning of development of hypertension as well as a risk for cardiovascular disease, heart attack and stroke. In the UK, non-pharmacological interventions are recommended for prehypertension management, but no reviews have focused on the effectiveness of these types of interventions solely in people with prehypertension. Therefore, the proposed systematic review will assess the clinical- and cost–effectiveness of non-pharmacological interventions in reducing or maintaining blood pressure in pre-hypertensive people.

Methods and analysis

This systematic review will follow the Preferred Reporting Items for Systematic Reviews and Meta-Analyses guidelines. The databases/ trial registries that will be searched to identify relevant randomised controlled trials (RCTs) and economic evaluations include Medline, EMBASE, CINAHL, PsycINFO, CENTRAL, the World Health Organization International Clinical Trials Registry Platform, ClinicalTrials.gov, Cochrane Library, Scopus, and the International HTA Database. Search terms have been identified by the team including an information specialist. Three reviewers will be involved in the study selection process. Risk of bias will be evaluated using the Cochrane risk-of-bias tool for RCTs, and the Consensus Health Economic Criteria list for economic evaluations. Findings from the included studies will be tabulated and synthesised narratively. Heterogeneity will be assessed

For peer review only - http://bmjopen.bmj.com/site/about/guidelines.xhtml

through visual inspection of forest plots and the calculation of the Chi^2 and I^2 statistics, and causes of heterogeneity will be assessed where sufficient data are available. If possible, we plan to investigate differential effects on specific sub-groups and from different types of interventions using meta-regression. Where relevant, the Grading of Recommendations, Assessment, Development, and Evaluations (GRADE) will be used to assess the certainty of the evidence found.

Ethics and dissemination

Ethical approval is not needed. Results will be published in a peer-reviewed journal, disseminated via the wider study website and shared with the study sites and participants.

Registration details: The review is registered with Prospero (CRD420232433047)

KEYWORDS: Prehypertension; blood pressure; non-pharmacological interventions

Strengths and limitations of this study

- To our knowledge, the systematic review will be the first to provide an up-to-date synthesis on non-pharmacological interventions to reduce blood pressure in individuals in the prehypertensive range.
- A comprehensive search strategy has been designed collaboratively with a skilled information specialist.
- There will be no restrictions applied to the searches regarding language or date of publication.
- This review will only include randomised controlled trials and comparative economic evaluations. Other study designs will be excluded.

INTRODUCTION

People with blood pressure (BP) in the prehypertension (PHT) range (120-139/80-89 mmHg), are at increased risk, compared to those with normal BP, of developing hypertension[1,2] and other cardiovascular disease (CVD)-related conditions, independent of progression to hypertension.[3-9] The estimated number of people with PHT is substantial, with around 40% of adults attending primary care clinics falling into this category.[10] BP exists on a continuum following a normal distribution, with risk of CVD increasing as BP increases. PHT, also known as “high-normal”, “elevated” or “raised” BP, lies between normal BP (<120/80 mmHg) and hypertension (>=140/90 mmHg).

PHT itself however is not regarded as an illness, rather it is a warning of “an insidious progression” of BP to problematic levels.[11] It is starting to be recognised that prevention needs to be at the heart of future healthcare[12] with a change of emphasis from fixing ill-health to protecting good health. PHT can be a useful sign to identify and alert those at-risk of developing hypertension and CVD, so they can take action to prevent, delay or reduce progression to disease status.[11]

In the UK, PHT does not require drug intervention like in hypertension. Rather, guidance[13] recommends that PHT management should be focused on lifestyle modification.

For peer review only - http://bmjopen.bmj.com/site/about/guidelines.xhtml

There is a plethora of primary research studies demonstrating that BP in people with PHT can be managed via various lifestyle interventions, including both single component interventions e.g., exercise,[14-16] diet,[17,18] breathing exercises,[19,20] yoga,[21,22] etc, as well as multi-component lifestyle interventions.[23,24]

Research has also demonstrated that lifestyle modifications at this “pre-risk” stage could provide lifetime benefits; for example, by making lifestyle changes, people with PHT could significantly reduce both their risk of developing hypertension[25] and also reverse PHT to normotensive levels.[26]

There are a number of published systematic reviews on this topic. However, these tend to focus on one type of non-pharmacological intervention such as exercise.[27] There are two existing reviews that assess the effectiveness of various non-pharmacological interventions on PHT.[28,29] However, both reviews included studies combining PHT and hypertensive participants making it impossible to determine what might be effective for those specifically with PHT. Additionally, neither included a cost-effectiveness review, and the most recent review[28] only looked at outcomes at 4-weeks, which therefore does not inform us about the longer-term effectiveness of non-pharmacological interventions.

Therefore, an up-to date review of current evidence on clinical- and cost-effectiveness of non-pharmacological interventions specifically for PHT would help guide research and practice in this area., with the potential to prevent not only progression to hypertension but also prevent CVD in the future. The aim of this review is to assess the clinical and cost-effectiveness of different non-pharmacological interventions for reducing BP and maintaining BP in adults with PHT.

Review question

What is the clinical- and cost-effectiveness of non-pharmacological interventions for reducing and/or maintaining blood pressure in adults with prehypertension, compared to usual care, or other non-pharmacological interventions?

METHODS AND ANALYSIS

Study Design

This systematic review will be conducted and reported following the Preferred Reporting Items for Systematic Reviews and Meta-Analyses (PRISMA) guidelines.

Search Strategy

Separate searches to identify randomised controlled trials (RCTs) or economic evaluations will be conducted. Seven databases and trial registries will be searched to identify relevant RCTs: Medline, EMBASE, CINAHL, PsycINFO, Cochrane Central Register of Controlled Trials (CENTRAL), the World Health Organization International Clinical Trials Registry Platform, and ClinicalTrials.gov. Medline, EMBASE, CINAHL, PsycINFO, Cochrane Library via Wiley (all databases), Scopus, and the International HTA Database will be searched to identify economic evaluations. We will screen the reference lists of all included studies and relevant review articles for any additional studies. There will be no restrictions applied to the searches regarding language or date of publication.

For peer review only - http://bmjopen.bmj.com/site/about/guidelines.xhtml

The search terms were identified by the team (including an information specialist) via an iterative process. The search strategies include keywords and subject headings relating to prehypertension which were combined with the Cochrane Highly Sensitive Search Strategy[30] for identifying Randomized Trials in Medline (sensitivity-maximising version) and the NHS EED Search Filter[31] to identify economic evaluations. The search strategies were adapted for use in each database and combined with database-specific search filters for RCTs and economic evaluations where these are available. The searches were conducted in May 2022. The search strategies for Medline can be found in Appendix 1.

Study Selection

Search results will be imported into EndNote (version X9, Clarivate Analytics, Philadelphia, PA), where duplicates will be removed. The final list for screening will then be imported into Rayyan®. Screening for the studies of clinical effectiveness and cost-effectiveness will be done as two separate processes. One reviewer will screen all the titles and abstracts of the search results to identify relevant articles, and a second reviewer will review at least 10% to ensure consistency. A third reviewer will then check those studies deemed as potentially eligible for the next stage. One reviewer will then independently screen the full texts of the potentially eligible articles, and a second reviewer will check the ones selected for inclusion, to determine final inclusion in the review. Disagreements will be resolved via discussion, and where necessary through the involvement of a third reviewer. The study selection process will be presented using a PRISMA flow diagram.

Inclusion Criteria

Only RCTs and comparative economic evaluations (including costs and/or consequences) will be eligible for inclusion in the review.

Condition or domain being studied

Prehypertension (PHT).

Studies that report data from samples with PHT AND hypertension (controlled or uncontrolled) will be included *only* if the data can be separated out, so that only data regarding PHT can be extracted.

Participants/population

Inclusion criteria: People aged 18 years old or older with blood pressure in the prehypertension range defined as systolic blood pressure 120-139mmHg and/or diastolic blood pressure 80-89 mmHg.

Exclusion criteria: We will exclude data from participants that have/had a diagnosis of hypertension, are on anti-hypertensive medication, are pregnant, have a life limiting disease or have previously had a cardiovascular event (e.g., heart attack, stroke, transient ischaemic attack) or have a pre-existing cardiovascular disease.

Intervention(s), exposure(s)

For peer review only - http://bmjopen.bmj.com/site/about/guidelines.xhtml

All non-pharmacological interventions will be considered, regardless of presentation or delivery mode (e.g., face-to-face, group, one-to-one, self-directed, etc), for inclusion. This will include investigations of foods and/ or compounds found in foods (e.g., caffeine, flavonoids).

Comparator(s)/control

Studies will be included if they compare non-pharmacological interventions to usual care, or if they compare different types of non-pharmacological interventions to each other.

Context

Study setting may be primary or secondary care, or community settings.

Main outcome(s)

Change in BP (both systolic and diastolic) between intervention and comparator will be the main outcome for the clinical effectiveness strand of our review, and studies that report such change will be included. BP could be office, ambulatory or self-monitored readings. We will also include studies that report a change in cardiovascular morbidity (cardiovascular disease, hypertension, myocardial infarction, or stroke/transient ischaemic attack),

For the cost-effectiveness strand the main outcomes will be a change in costs, quality of life (QoL) and cost-effectiveness, and studies reporting these will be included.

All follow-up lengths will be included, with the differential effects associated with different durations explored in sub-group analyses.

Measures of effect

The measures of effect for change in BP and change in QoL will be the mean difference, standard deviation, and 95% confidence interval (CI).

For occurrence of CVD, hypertension, stroke, TIA, and MI, the measure of effect will be the odds ratio (OR).

For cost-effectiveness, we will consider mean differences in costs and QoL between comparators (and any dispersion measures as reported by the included studies), as well as cost per marginal gain in the specific QoL measures employed by the studies (e.g., quality-adjusted life years or QALYs).

Data extraction (selection and coding)

Two reviewers will independently extract data from the agreed eligible studies. A coding scheme will be developed and piloted to ensure all relevant data is captured and to ensure both reviewers are extracting data in a standardised manner. It is envisaged the following data will be extracted:

- *Study context:* authors, study title, journal, year of publication, location of study, funding details, conflicts of interest;

For peer review only - http://bmjopen.bmj.com/site/about/guidelines.xhtml

- *Design and Methods:* method of randomisation, details of study sites (number, location, setting etc), variables of interest, data collection tools, planned follow-up timings, key attributes of economic evaluations (e.g., perspective, design, time horizon, characteristics of cost and outcome data, cost-effectiveness measure employed);
- *Participants:* number, basic demographics (age, gender, ethnicity), blood pressure, co-morbidities;
- *Interventions:* type of intervention, details of the intervention including length, frequency and duration, comparison, mode of delivery, provider, content and components;
- *Outcomes:* systolic blood pressure, diastolic blood pressure, presence of cardiovascular morbidity (hypertension, MI, stroke, TIA, cardiovascular disease), costs, QoL and cost-effectiveness at baseline and the stated follow-up time points of each study.

For peer review only

Risk of bias (quality) assessment

Two reviewers will independently critically appraise the eligible studies. Risk of bias will be evaluated using the Cochrane risk-of-bias tool (RoB1) for RCTs, and the Consensus Health Economic Criteria (CHEC) list for economic evaluations.[32] Risk of bias will be incorporated within the synthesis and interpretation of the evidence, using Grading of Recommendations, Assessment, Development, and Evaluations (GRADE) framework.[33]

Strategy for data synthesis

Studies will be synthesised through narrative reviews with tabulation of results of included studies. Where possible, treatment effects for all comparisons and outcomes will be synthesised through meta-analyses, with the approach taken dependant on the outcome assessed and the data available. Dichotomous data will be presented as risk ratios with 95% CIs. Continuous data will be synthesised as weighted mean differences (MD) when outcomes are assessed on the same scale, or standardised weighted mean difference (SMD) when different scales are used to measure the same underlying construct, with 95% CI. Where the outcomes represent time-to-event data (e.g., overall survival), the (log) hazard ratio with 95% CI will be used as the summary measure. Heterogeneity will be assessed through visual inspection of forest plots and the calculation of the Chi^2 and I^2 statistics. Causes of heterogeneity will be assessed where sufficient data is available, including factors such as participant characteristics (e.g., age, sex), baseline BP, method of obtaining BP readings (office, ambulatory, home), duration of intervention, co-morbidities and delivery/ mode of intervention. Where appropriate, these will be investigated further through sub-group analyses and through meta-regression. Sensitivity analyses will explore possible causes of methodological heterogeneity, where sufficient data are available. This would include assessing the effects of studies that may be affected by factors such as risk of bias associated with allocation concealment, high loss to follow-up or lack of blinding in assessment of outcomes. Where data are missing, particularly measures of variation (e.g., standard deviation), we will contact study authors or impute values. It is likely that the analysis will focus on direct comparisons of intervention effects through pairwise meta-analyses. Where evidence allows, we will consider conducting network meta-analysis through both direct and indirect evidence within connected networks of trials. Pairwise meta-analyses of direct comparisons will be conducted using STATA v17 (version 17, StataCorp, Texas, USA) or Comprehensive Meta-analysis v4, while NMAs will be estimated using the WinBUGS software (version 1.4.3) (MRC Biostatistics Unit, Cambridge, UK)(http://www.mrc-bsu.cam.ac.uk/bugs/winbugs/contents.shtml).

Analysis of subgroups or subsets

For peer review only - http://bmjopen.bmj.com/site/about/guidelines.xhtml

Depending on the variety of non-pharmacological interventions and studies' populations and on the available data, we plan to investigate differential effects on specific sub-groups (e.g., by age, sex, BP levels, co-morbidities, health-related quality of life), and from different types of interventions (e.g., by duration, setting, mode of delivery) using meta-regression. Where this quantitative approach is not possible, we will discuss the sub-group effects narratively.

Assessment of certainty of evidence

Where relevant, we will assess the level of certainty of the evidence found in the included studies using the GRADE framework.[21]

PATIENT AND PUBLIC INVOLVEMENT

Patients and the public were not directly involved in designing this systematic review protocol. The systematic review is connected to a larger programme of work called REVERSE (Risk Education InterVEntion for Raised blood preSsurE). REVERSE aims to investigate the feasibility of blood pressure self-monitoring for people with prehypertension, which was co-produced with members of the public with prehypertension and hypertension. The results of both the systematic review of non-pharmacological interventions and feasibility study of self-monitoring will help with co-producing future lifestyle interventions, with the aim of preventing hypertension and associated conditions, which will be tested in future effectiveness studies.

ETHICS AND DISSEMINATION

Ethical approval is not needed for this systematic review. The review is registered with Prospero (CRD420232433047), and any important protocol amendments will be updated on the record. It is intended to publish the completed review in a peer-reviewed journal. Results will also be disseminated via the REVERSE study website and will be shared with the REVERSE study sites and participants.

TWITTER

@ReverseStudy

WEBSITE

www.reversestudy.co.uk

AUTHORS CONTRIBUTIONS

EB was responsible for the conception, design and writing of the manuscript. LH, JS, EB, RG, CW, VB, NW, PR and AC contributed to the conception and design. EB, LH, VB and NI contributed to the writing of the manuscript. CH designed the search strategy and performed the preliminary database searches. All authors read and approved the final manuscript. EB is the guarantor of the review.

For peer review only - http://bmjopen.bmj.com/site/about/guidelines.xhtml

FUNDING STATEMENT

EB holds a NIHR RfPB grant (NIHR201028). This work is related to this funding but is not directly funded by it. The views expressed are those of the author(s) and not necessarily those of the NIHR or the Department of Health and Social Care.

COMPETING INTERESTS STATEMENT

VB, CH, AC, JS and CW are part funded by the National Institute for Health Research Applied Research Collaboration North West Coast (NIHR ARC NWC). The views expressed are those of the authors and not necessarily those of the NHS, the NIHR, or the Department of Health and Social Care.

For peer review only

REFERENCES

[1] Wang Q, Wang Z, Yao W, Wu X, Huang J, Huang L, et al. Anthropometric indices predict the development of hypertension in normotensive and pre- hypertensive middle-aged women in Tianjin, China: A prospective cohort study. *Medical Science Monitor.* 2018;24:1871-9.

[2] Zheng L, Sun Z, Zhang X, Xu C, Li J, Hu D, et al. Predictors of progression from prehypertension to hypertension among rural Chinese adults: Results from Liaoning Province. *European Journal of Cardiovascular Prevention and Rehabilitation*. 2010;17(2):217-22.

[3] Huang Y, Su L, Cai X, Mai W, Wang S, Hu Y, et al. Association of all-cause and cardiovascular mortality with prehypertension: A meta-analysis. *American Heart Journal*. 2014;167(2):160-8.e1.

[4] Guo X, Yingxian S. Quantitative association between prehypertension and different types of cardiovascular outcomes and diabetes: A systematic review and meta-analysis of prospective studies worldwide. *Heart*. 2013;3:A115.

[5] Li Y, Xia P, Xu L, Wang Y, Chen L. A Meta-analysis on prehypertension and chronic kidney disease. *PLoS ONE*. 2016;11(6):e0156575.

[6] Kanno A, Kikuya M, Ohkubo T, Hashimoto T, Satoh M, Hirose T, et al. Pre-hypertension as a significant predictor of chronic kidney disease in a general population: The Ohasama Study. *Nephrology Dialysis Transplantation*. 2012;27(8):3218-23.

[7] Huang Y, Cai X, Liu C, Zhu D, Hua J, Hu Y, et al. Prehypertension and the risk of coronary heart disease in Asian and western populations: A meta-analysis. *Journal of the American Heart Association*. 2015;4(2):e001519.

[8] Lee M, Saver JL, Chang B, Chang KH, Lin K, Ovbiagele B. Baseline pre-hypertension and risk of incident stroke: A meta-analysis. *Stroke*. 2011;42(3):e49-e50.

[9] Winegarden CR. From “prehypertension” to hypertension? Additional evidence. *Annals of Epidemiology*. 2005;15(9):720-5.

[10] Joffres M, Falaschetti E, Gillespie C, Robitaille C, Loustalot F, Poulter N, et al. Hypertension prevalence, awareness, treatment and control in national surveys from England, the USA and Canada, and correlation with stroke and ischaemic heart disease mortality: a cross-sectional study. *BMJ Open*. 2013;3:e003423.

[11] Collier SR, Landram MJ. Treatment of prehypertension: lifestyle and/or medication. *Vascular Health and Risk Management.* 2012;8:613-19.

[12] National Health Service. *The NHS Long Term Plan*. Available at: https://www.longtermplan.nhs.uk/publication/nhs-long-term-plan/ [Accessed 19th February 2019].

[13] Whelton PK, Carey RM, Aronow WS, Casey DE Jr, Collins KJ, Dennison Himmelfarb C, et al. 2017 ACC/AHA/AAPA/ABC/ACPM/AGS/APhA/ASH/ASPC/NMA/PCNA guideline for the prevention, detection, evaluation, and management of high blood pressure in adults: executive summary: a report of the American College of Cardiology/American Heart Association Task Force on Clinical Practice Guidelines. *Hypertension*. 2018;71:e13-e115.

[14] Duangpunmat U, Kalampakorn S, Pichayapinyo P. An effect of walking exercise applying the theory of planned behavior in people at risk of hypertension. J Med Assoc Thai. . 2013, 96 Suppl 5

[15] Azadpour N, Tartibian B, Kosar SN. Effects of aerobic exercise training on ACE and ADRB2 gene expression, plasma angiotensin II level, and flow-mediated dilation: a study on obese postmenopausal women with prehypertension. Menopause. . 2017;24(3):269-277.

[16] Moore LL, Visioni AJ, Qureshi MM, Bradlee ML, Ellison RC, D-Agostino R. Weight loss in overweight adults and the long-term risk of hypertension: The Framingham Study. *Archives of Internal Medicine*. 2005;165:1298–303.

For peer review only - http://bmjopen.bmj.com/site/about/guidelines.xhtml

17 Dorough AE, Winett RA, Anderson ES. Davy BM, Martin EC, Hedrick V. DASH to wellness: emphasizing self-regulation through e-health in adults with prehypertension. Health psychology. 2014; 33 (3): 249-54.

18 Rahimi ARO, Mhmoodpoor A, Sanaie S. The effect of high-calcium and high-potassium diet on grade-I hypertension and high normal blood pressure. Pakistan Journal of Medical Sciences. 2007; 23 (4); 589-592.

19 Wang SZ, Li S, Xu XY, Lin GP, Shao L, Zhao Y, Wang TH. Effect of slow abdominal breathing combined with biofeedback on blood pressure and heart rate variability in prehypertension. Journal of alternative and complementary medicine. 2010. 16 (10); 1039-45.

20 Abdullah A, Bakri S, Rasyid H, Aman M, Kasim H, Harjianti T, dan Tendean P, Seweng A. Effects of deep breath on blood pressure in normo-prehypertensive subjects. European Journal of Molecular and Clinical Medicine. 2021; 8 (2): 1636-1642.

21 Singh, V. P.; Kh; elwal, B.; Effectiveness Of Yoga and Lifestyle Modification On Prehypertensive Subjects-A Randomised Controlled Trial. NeuroQuantology. 2022. 20 (17); 1323-1337.

22 Ankolekar VH, Reddy GG, Sanju CSV, Mamatha H. Role of yoga intervention on quality of life and prehypertension. *Indian J Tradit Know.* 2019: 18:351–5.

23 He, Jiang[a,b]; Streiffer, Richard H[c]; Muntner, Paul[a,b]; Krousel-Wood, Marie A[c]; Whelton, Paul K[a,b]. Effect of dietary fiber intake on blood pressure: a randomized, double-blind, placebo-controlled trial. Journal of Hypertension 22(1):p 73-80, January 2004.

24 Svetkey LP, Erlinger TP, Vollmer WM, Feldstein A, Cooper LS, Appel LJ, Ard JD, Elmer PJ, Harsha D, Stevens VJ. Effect of lifestyle modifications on blood pressure by race, sex, hypertension status, and age, Journal of human hypertension. 2005: 19 (1); 21-31.

25 Chiu YH, Wu SC, Tseng CD, Yen MF, Chen TH. Progression of pre-hypertension, stage 1 and 2 hypertension (JNC 7): a population-based study in Keelung, Taiwan (Keelung Community-based Integrated Screening No. 9). Journal of Hypertension. 2006;24(5):821-8.

26 Padilla J, Wallace JP, Park S. Accumulation of physical activity reduces blood pressure in pre- and hypertension. Medicine and Science in Sports and Exercise. 2005;37:1264–75.

27 Mehrnoosh E, Atefeh A, Sahar V, Majid G, Iqbal DR, Yamuna S. Effect of Different Types of Aerobic Exercise on Individuals With and Without Hypertension: An Updated Systematic Review. Current problems in cardiology. 2023: 48 (3); 101034.

28 Shao T, Liang L, Zhou C, Tang Y, Gao W, Tu Y, Yin Y, Malone DC, Tang W. Short-term efficacy of non-pharmacological interventions for global population with elevated blood pressure: A network meta-analysis Frontiers in public health. 2022. 10; 1051581.

29 Fu J, Liu Y, Zhang L, Zhou L, Li D, Quan H, et al. Nonpharmacologic Interventions for Reducing Blood Pressure in Adults With Prehypertension to Established Hypertension. *Journal of the American Heart Association.* 2020;9(19):e016804.

30 Lefebvre C, Glanville J, Briscoe S, Featherstone R, Littlewood A, Marshall C, et al. Chapter 4: Searching for and selecting studies. In: Higgins JPT, Thomas J, Chandler J, Cumpston M, Li T, Page MJ, Welch VA (editors). *Cochrane Handbook for Systematic Reviews of Interventions* version 6.3 (updated February 2022). Cochrane, 2022. Available: www.training.cochrane.org/handbook.

31 University of York Centre for Reviews and Dissemination. How are studies identified for NHS EED. 2009. Available: https://www.crd.york.ac.uk/crdweb/searchstrategies.asp [Accessed 15 May 2023].

32 Evers S, Goossens M, De Vet H, Van Tulder M, Ament A. Criteria list for assessment of methodological quality of economic evaluations: Consensus on Health Economic Criteria. *International Journal of Technology Assessment in Health Care*. 2005;21(2):240-245.

For peer review only

For peer review only - http://bmjopen.bmj.com/site/about/guidelines.xhtml

[33] Schünemann HJ, Higgins JPT, Vist GE, Glasziou P, Akl EA, Skoetz N, Guyatt GH; on behalf of the Cochrane GRADEing Methods Group and the Cochrane Statistical Methods Group. Cochrane Handbook for Systematic Reviews of Interventions version 6.3, Section 14.3. 2022. Available: https://training.cochrane.org/handbook/current/chapter-14#section-14-3.

For peer review only

Non-Pharmacological intervention for PHT systematic review

Medline Search Strategy

Search conducted May 26th 2023

Search Number	Query	Hits
1	Prehypertension/	1144
2	prehypertensi*.ti,ab,kw,kf.	2946
3	pre-hypertensi*.ti,ab,kw,kf.	925
4	(pre-htn or prehtn).tw.	113
5	((stage 1 or stage one) adj hypertensi*).tw.	666
6	(elevated blood pressure or elevated bp).ti.	866
7	high-normal blood pressure.tw.	409
8	raised blood pressure.ti.	83
9	borderline hypertensi*.tw.	1403
10	early hypertensi*.tw.	365
11	raised systolic blood pressure.tw.	75
12	raised diastolic blood pressure.tw.	16
13	("at risk" adj1 hypertensi*).tw.	43
14	((("120" adj2 "129") or ("130" adj2 "139") or ("120" adj2 "139") or ("80" adj2 "89")) and (blood pressure or bp)).tw.	964
15	or/1-14	7975
16	exp randomized controlled trial/	594769
17	controlled clinical trial.pt.	95314
18	randomi#ed.ab.	721331
19	placebo.ab.	238387
20	randomly.ab.	408822
21	trial.ab.	649200
22	groups.ab.	2520111
23	or/16-22	3638767
24	exp animals/ not humans.sh.	5123796
25	23 not 24	3135655
26	15 and 25	2372

For peer review only

Reporting checklist for protocol of a systematic review and meta analysis.

Based on the PRISMA-P guidelines.

Instructions to authors

Complete this checklist by entering the page numbers from your manuscript where readers will find each of the items listed below.

Your article may not currently address all the items on the checklist. Please modify your text to include the missing information. If you are certain that an item does not apply, please write "n/a" and provide a short explanation.

Upload your completed checklist as an extra file when you submit to a journal.

In your methods section, say that you used the PRISMA-Preporting guidelines, and cite them as:

Moher D, Shamseer L, Clarke M, Ghersi D, Liberati A, Petticrew M, Shekelle P, Stewart LA. Preferred Reporting Items for Systematic Review and Meta-Analysis Protocols (PRISMA-P) 2015 statement. Syst Rev. 2015;4(1):1.

		Reporting Item	Page Number
**Title**			
Identification	#1a	Identify the report as a protocol of a systematic review	1
Update	#1b	If the protocol is for an update of a previous systematic review, identify as such	n/a
**Registration**			
	#2	If registered, provide the name of the registry (such as 7PROSPERO) and registration number	2 & 7
**Authors**			
Contact	#3a	Provide name, institutional affiliation, e-mail address of all protocol authors; provide physical mailing address of corresponding author	1
Contribution	#3b	Describe contributions of protocol authors and identify the guarantor of the review	7

For peer review only

**Amendments**			
	#4	If the protocol represents an amendment of a previously completed or published protocol, identify as such and list changes; otherwise, state plan for documenting important protocol amendments	7
**Support**			
Sources	#5a	Indicate sources of financial or other support for the review	8
Sponsor	#5b	Provide name for the review funder and / or sponsor	8
Role of sponsor or funder	#5c	Describe roles of funder(s), sponsor(s), and / or institution(s), if any, in developing the protocol	8
**Introduction**			
Rationale	#6	Describe the rationale for the review in the context of what is already known	3
Objectives	#7	Provide an explicit statement of the question(s) the review will address with reference to participants, interventions, comparators, and outcomes (PICO)	3
**Methods**			
Eligibility criteria	#8	Specify the study characteristics (such as PICO, study design, setting, time frame) and report characteristics (such as years considered, language, publication status) to be used as criteria for eligibility for the review	4-5
Information sources	#9	Describe all intended information sources (such as electronic databases, contact with study authors, trial registers or other grey literature sources) with planned dates of coverage	4
Search strategy	#10	Present draft of search strategy to be used for at least one electronic database, including planned limits, such that it could be repeated	Appendix 1
Study records - data management	#11a	Describe the mechanism(s) that will be used to manage records and data throughout the review	4
Study records - selection process	#11b	State the process that will be used for selecting studies (such as two independent reviewers) through each phase of the review (that is, screening, eligibility and inclusion in meta-analysis)	4
Study records - data	#11c	Describe planned method of extracting data from reports (such as	5-6

For peer review only

collection process		piloting forms, done independently, in duplicate), any processes for obtaining and confirming data from investigators	
Data items	#12	List and define all variables for which data will be sought (such as PICO items, funding sources), any pre-planned data assumptions and simplifications	6
Outcomes and prioritization	#13	List and define all outcomes for which data will be sought, including prioritization of main and additional outcomes, with rationale	6
Risk of bias in individual studies	#14	Describe anticipated methods for assessing risk of bias of individual studies, including whether this will be done at the outcome or study level, or both; state how this information will be used in data synthesis	6
Data synthesis	#15a	Describe criteria under which study data will be quantitatively synthesised	6-7
Data synthesis	#15b	If data are appropriate for quantitative synthesis, describe planned summary measures, methods of handling data and methods of combining data from studies, including any planned exploration of consistency (such as I2, Kendall's τ)	6-7
Data synthesis	#15c	Describe any proposed additional analyses (such as sensitivity or subgroup analyses, meta-regression)	6-7
Data synthesis	#15d	If quantitative synthesis is not appropriate, describe the type of summary planned	n/a
Meta-bias(es)	#16	Specify any planned assessment of meta-bias(es) (such as publication bias across studies, selective reporting within studies)	6
Confidence in cumulative evidence	#17	Describe how the strength of the body of evidence will be assessed (such as GRADE)	6

None The PRISMA-P elaboration and explanation paper is distributed under the terms of the Creative Commons Attribution License CC-BY. This checklist can be completed online using https://www.goodreports.org/, a tool made by the EQUATOR Network in collaboration with Penelope.ai

For peer review only

BMJ Open

Non-pharmacological interventions for the reduction and maintenance of blood pressure in people with prehypertension: A systematic review protocol

Journal:	*BMJ Open*
Manuscript ID	bmjopen-2023-078189.R2
Article Type:	Protocol
Date Submitted by the Author:	21-Dec-2023
Complete List of Authors:	Bray, Emma; University of Central Lancashire, Senior Research Fellow, Stroke Research Team, School of Nursing<br>Georgiou, Rachel; University of Central Lancashire, Senior Research Fellow, Stroke Research Team, School of Nursing<br>Hives, Lucy; University of Central Lancashire, Research Associate, School of Community Health and Midwifery<br>Iqbal, Nafisa; University of Central Lancashire, Stroke Team<br>Benedetto, Valerio; University of Central Lancashire, Research Fellow, Synthesis, Economic Evaluation and Decision Science Group<br>Spencer, Joseph; University of Central Lancashire, Senior Research Assistant, Research Facilitation and Delivery Unit<br>Harris, Cath; University of Central Lancashire, Information Specialist, Synthesis, Economic Evaluation and Decision Science Group<br>Clegg, Andrew; University of Central Lancashire, Professor of Health Services Research, Synthesis, Economic Evaluation and Decision Science Group<br>Williams, Nefyn; University of Liverpool, Professor of Primary Care, Department of Primary Care and Mental Health<br>Rutter, Paul; University of Portsmouth, Professor of Pharmacy Practice, School of Biomedical Sciences<br>Watkins, Caroline; University of Central Lancashire, Professor of Stroke and Older People's Care, Stroke Research Team
**Primary Subject Heading**:	Public health
Secondary Subject Heading:	Cardiovascular medicine, Health economics
Keywords:	Blood Pressure, Prehypertension, Primary Prevention, Systematic Review

SCHOLARONE™
Manuscripts

Non-pharmacological interventions for the reduction and maintenance of blood pressure in people with prehypertension: A systematic review protocol

CORRESPONDING AUTHOR: Dr Emma Bray, EBray@uclan.ac.uk

Authors

Dr Emma Bray[1], Rachel Georgiou[1], Lucy Hives[2], Nafisa Iqbal[1], Dr Valerio Benedetto[3], Joseph Spencer[4], Catherine Harris[3], Prof Andrew Clegg[3], Prof Nefyn Williams[5], Prof Paul Rutter[6], Prof Caroline Watkins[1]

1. Stroke Research Team, School of Nursing, Faculty of Health and Care, University of Central Lancashire, PR1 2HE, UK.
2. School of Community Health and Midwifery, University of Central Lancashire, UK.
3. Synthesis, Economic Evaluation and Decision Science Group; Research Fellow, Health Technology Assessment Unit, University of Central Lancashire, UK.
4. Research Facilitation and Delivery Unit, University of Central Lancashire, UK.
5. Department of Primary Care and Mental Health, Institute of Population Health, University of Liverpool, UK.
6. School of Biomedical Sciences, University of Portsmouth, Portsmouth, UK.

Word count: 2277

ABSTRACT

Introduction

Prehypertension is defined as blood pressure that is above the normal range but not high enough to be classed as hypertension. Prehypertension is a warning of development of hypertension as well as a risk for cardiovascular disease, heart attack and stroke. In the UK, non-pharmacological interventions are recommended for prehypertension management, but no reviews have focused on the effectiveness of these types of interventions solely in people with prehypertension. Therefore, the proposed systematic review will assess the clinical- and cost–effectiveness of non-pharmacological interventions in reducing or maintaining blood pressure in pre-hypertensive people.

Methods and analysis

This systematic review will follow the Preferred Reporting Items for Systematic Reviews and Meta-Analyses guidelines. The databases/ trial registries that will be searched to identify relevant randomised controlled trials (RCTs) and economic evaluations include Medline, EMBASE, CINAHL, PsycINFO, CENTRAL, the World Health Organization International Clinical Trials Registry Platform, ClinicalTrials.gov, Cochrane Library, Scopus, and the International HTA Database. Search terms have been identified by the team including an information specialist. Three reviewers will be involved in the study selection process. Risk of bias will be evaluated using the Cochrane risk-of-bias tool for RCTs, and the Consensus Health Economic Criteria list for economic evaluations. Findings from the included studies will be tabulated and synthesised narratively. Heterogeneity will be assessed

For peer review only - http://bmjopen.bmj.com/site/about/guidelines.xhtml

through visual inspection of forest plots and the calculation of the Chi^2 and I^2 statistics, and causes of heterogeneity will be assessed where sufficient data are available. If possible, we plan to investigate differential effects on specific sub-groups and from different types of interventions using meta-regression. Where relevant, the Grading of Recommendations, Assessment, Development, and Evaluations (GRADE) will be used to assess the certainty of the evidence found.

Ethics and dissemination

Ethical approval is not needed. Results will be published in a peer-reviewed journal, disseminated via the wider study website and shared with the study sites and participants.

Registration details: The review is registered with Prospero (CRD420232433047)

KEYWORDS: Prehypertension; blood pressure; non-pharmacological interventions

Strengths and limitations of this study

- To our knowledge, the systematic review will be the first to provide an up-to-date synthesis on non-pharmacological interventions to reduce blood pressure in individuals in the prehypertensive range.
- A comprehensive search strategy has been designed collaboratively with a skilled information specialist.
- There will be no restrictions applied to the searches regarding language or date of publication.
- This review will only include randomised controlled trials and comparative economic evaluations. Other study designs will be excluded.

INTRODUCTION

People with blood pressure (BP) in the prehypertension (PHT) range (120-139/80-89 mmHg), are at increased risk, compared to those with normal BP, of developing hypertension[1,2] and other cardiovascular disease (CVD)-related conditions, independent of progression to hypertension.[3-9] The estimated number of people with PHT is substantial, with around 40% of adults attending primary care clinics falling into this category.[10] BP exists on a continuum following a normal distribution, with risk of CVD increasing as BP increases. PHT, also known as “high-normal”, “elevated” or “raised” BP, lies between normal BP (<120/80 mmHg) and hypertension (>=140/90 mmHg).

PHT itself however is not regarded as an illness, rather it is a warning of “an insidious progression” of BP to problematic levels.[11] It is starting to be recognised that prevention needs to be at the heart of future healthcare[12] with a change of emphasis from fixing ill-health to protecting good health. PHT can be a useful sign to identify and alert those at-risk of developing hypertension and CVD, so they can take action to prevent, delay or reduce progression to disease status.[11]

In the UK, PHT does not require drug intervention like in hypertension. Rather, guidance[13] recommends that PHT management should be focused on lifestyle modification.

For peer review only - http://bmjopen.bmj.com/site/about/guidelines.xhtml

There is a plethora of primary research studies demonstrating that BP in people with PHT can be managed via various lifestyle interventions, including both single component interventions e.g., exercise,[14-16] diet,[17,18] breathing exercises,[19,20] yoga,[21,22] etc, as well as multi-component lifestyle interventions.[23,24]

Research has also demonstrated that lifestyle modifications at this “pre-risk” stage could provide lifetime benefits; for example, by making lifestyle changes, people with PHT could significantly reduce both their risk of developing hypertension[25] and also reverse PHT to normotensive levels.[26]

There are a number of published systematic reviews on this topic. However, these tend to focus on one type of non-pharmacological intervention such as exercise.[27] There are two existing reviews that assess the effectiveness of various non-pharmacological interventions on PHT.[28,29] However, both reviews included studies combining PHT and hypertensive participants making it impossible to determine what might be effective for those specifically with PHT. Additionally, neither included a cost-effectiveness review, and the most recent review[28] only looked at outcomes at 4-weeks, which therefore does not inform us about the longer-term effectiveness of non-pharmacological interventions.

Therefore, an up-to date review of current evidence on clinical- and cost-effectiveness of non-pharmacological interventions specifically for PHT would help guide research and practice in this area., with the potential to prevent not only progression to hypertension but also prevent CVD in the future. The aim of this review is to assess the clinical and cost-effectiveness of different non-pharmacological interventions for reducing BP and maintaining BP in adults with PHT.

Review question

What is the clinical- and cost-effectiveness of non-pharmacological interventions for reducing and/or maintaining blood pressure in adults with prehypertension, compared to usual care, or other non-pharmacological interventions?

METHODS AND ANALYSIS

Study Design

This systematic review will be conducted and reported following the Preferred Reporting Items for Systematic Reviews and Meta-Analyses (PRISMA) guidelines.

Search Strategy

Separate searches to identify randomised controlled trials (RCTs) or economic evaluations will be conducted. Seven databases and trial registries will be searched to identify relevant RCTs: Medline, EMBASE, CINAHL, PsycINFO, Cochrane Central Register of Controlled Trials (CENTRAL), the World Health Organization International Clinical Trials Registry Platform, and ClinicalTrials.gov. Medline, EMBASE, CINAHL, PsycINFO, Cochrane Library via Wiley (all databases), Scopus, and the International HTA Database will be searched to identify economic evaluations. We will screen the reference lists of all included studies and relevant review articles for any additional studies. There will be no restrictions applied to the searches regarding language or date of publication.

For peer review only - http://bmjopen.bmj.com/site/about/guidelines.xhtml

The search terms were identified by the team (including an information specialist) via an iterative process. The search strategies include keywords and subject headings relating to prehypertension which were combined with the Cochrane Highly Sensitive Search Strategy[30] for identifying Randomized Trials in Medline (sensitivity-maximising version) and the NHS EED Search Filter[31] to identify economic evaluations. The search strategies were adapted for use in each database and combined with database-specific search filters for RCTs and economic evaluations where these are available. The searches were conducted in May 2023. The search strategies for Medline can be found in Appendix 1.

Study Selection

Search results will be imported into EndNote (version X9, Clarivate Analytics, Philadelphia, PA), where duplicates will be removed. The final list for screening will then be imported into Rayyan®. Screening for the studies of clinical effectiveness and cost-effectiveness will be done as two separate processes. One reviewer will screen all the titles and abstracts of the search results to identify relevant articles, and a second reviewer will review at least 10% to ensure consistency. A third reviewer will then check those studies deemed as potentially eligible for the next stage. One reviewer will then independently screen the full texts of the potentially eligible articles, and a second reviewer will check the ones selected for inclusion, to determine final inclusion in the review. Disagreements will be resolved via discussion, and where necessary through the involvement of a third reviewer. The study selection process will be presented using a PRISMA flow diagram.

Inclusion Criteria

Only RCTs and comparative economic evaluations (including costs and/or consequences) will be eligible for inclusion in the review.

Condition or domain being studied

Prehypertension (PHT).

Studies that report data from samples with PHT AND hypertension (controlled or uncontrolled) will be included *only* if the data can be separated out, so that only data regarding PHT can be extracted.

Participants/population

Inclusion criteria: People aged 18 years old or older with blood pressure in the prehypertension range defined as systolic blood pressure 120-139mmHg and/or diastolic blood pressure 80-89 mmHg.

Exclusion criteria: We will exclude data from participants that have/had a diagnosis of hypertension, are on anti-hypertensive medication, are pregnant, have a life limiting disease or have previously had a cardiovascular event (e.g., heart attack, stroke, transient ischaemic attack) or have a pre-existing cardiovascular disease.

Intervention(s), exposure(s)

For peer review only - http://bmjopen.bmj.com/site/about/guidelines.xhtml

For peer review only

All non-pharmacological interventions will be considered, regardless of presentation or delivery mode (e.g., face-to-face, group, one-to-one, self-directed, etc), for inclusion. This will include investigations of foods and/ or compounds found in foods (e.g., caffeine, flavonoids).

Comparator(s)/control

Studies will be included if they compare non-pharmacological interventions to usual care, or if they compare different types of non-pharmacological interventions to each other.

Context

Study setting may be primary or secondary care, or community settings.

Main outcome(s)

Change in BP (both systolic and diastolic) between intervention and comparator will be the main outcome for the clinical effectiveness strand of our review, and studies that report such change will be included. BP could be office, ambulatory or self-monitored readings. We will also include studies that report a change in cardiovascular morbidity (cardiovascular disease, hypertension, myocardial infarction, or stroke/transient ischaemic attack),

For the cost-effectiveness strand the main outcomes will be a change in costs, quality of life (QoL) and cost-effectiveness, and studies reporting these will be included.

All follow-up lengths will be included, with the differential effects associated with different durations explored in sub-group analyses.

Measures of effect

The measures of effect for change in BP and change in QoL will be the mean difference, standard deviation, and 95% confidence interval (CI).

For occurrence of CVD, hypertension, stroke, TIA, and MI, the measure of effect will be the odds ratio (OR).

For cost-effectiveness, we will consider mean differences in costs and QoL between comparators (and any dispersion measures as reported by the included studies), as well as cost per marginal gain in the specific QoL measures employed by the studies (e.g., quality-adjusted life years or QALYs).

Data extraction (selection and coding)

Two reviewers will independently extract data from the agreed eligible studies. A coding scheme will be developed and piloted to ensure all relevant data is captured and to ensure both reviewers are extracting data in a standardised manner. It is envisaged the following data will be extracted:

- *Study context:* authors, study title, journal, year of publication, location of study, funding details, conflicts of interest;

For peer review only

- *Design and Methods:* method of randomisation, details of study sites (number, location, setting etc), variables of interest, data collection tools, planned follow-up timings, key attributes of economic evaluations (e.g., perspective, design, time horizon, characteristics of cost and outcome data, cost-effectiveness measure employed);
- *Participants:* number, basic demographics (age, gender, ethnicity), blood pressure, co-morbidities;
- *Interventions:* type of intervention, details of the intervention including length, frequency and duration, comparison, mode of delivery, provider, content and components;
- *Outcomes:* systolic blood pressure, diastolic blood pressure, presence of cardiovascular morbidity (hypertension, MI, stroke, TIA, cardiovascular disease), costs, QoL and cost-effectiveness at baseline and the stated follow-up time points of each study.

Risk of bias (quality) assessment

Two reviewers will independently critically appraise the eligible studies. Risk of bias will be evaluated using the Cochrane risk-of-bias tool (RoB1) for RCTs, and the Consensus Health Economic Criteria (CHEC) list for economic evaluations.[32] Risk of bias will be incorporated within the synthesis and interpretation of the evidence, using Grading of Recommendations, Assessment, Development, and Evaluations (GRADE) framework.[33]

Strategy for data synthesis

Studies will be synthesised through narrative reviews with tabulation of results of included studies. Where possible, treatment effects for all comparisons and outcomes will be synthesised through meta-analyses, with the approach taken dependant on the outcome assessed and the data available. Dichotomous data will be presented as risk ratios with 95% CIs. Continuous data will be synthesised as weighted mean differences (MD) when outcomes are assessed on the same scale, or standardised weighted mean difference (SMD) when different scales are used to measure the same underlying construct, with 95% CI. Where the outcomes represent time-to-event data (e.g., overall survival), the (log) hazard ratio with 95% CI will be used as the summary measure. Heterogeneity will be assessed through visual inspection of forest plots and the calculation of the Chi^2 and I^2 statistics. Causes of heterogeneity will be assessed where sufficient data is available, including factors such as participant characteristics (e.g., age, sex), baseline BP, method of obtaining BP readings (office, ambulatory, home), duration of intervention, co-morbidities and delivery/ mode of intervention. Where appropriate, these will be investigated further through sub-group analyses and through meta-regression. Sensitivity analyses will explore possible causes of methodological heterogeneity, where sufficient data are available. This would include assessing the effects of studies that may be affected by factors such as risk of bias associated with allocation concealment, high loss to follow-up or lack of blinding in assessment of outcomes. Where data are missing, particularly measures of variation (e.g., standard deviation), we will contact study authors or impute values. It is likely that the analysis will focus on direct comparisons of intervention effects through pairwise meta-analyses. Where evidence allows, we will consider conducting network meta-analysis through both direct and indirect evidence within connected networks of trials. Pairwise meta-analyses of direct comparisons will be conducted using STATA v17 (version 17, StataCorp, Texas, USA) or Comprehensive Meta-analysis v4, while NMAs will be estimated using the WinBUGS software (version 1.4.3) (MRC Biostatistics Unit, Cambridge, UK)(http://www.mrc-bsu.cam.ac.uk/bugs/winbugs/contents.shtml).

Analysis of subgroups or subsets

For peer review only - http://bmjopen.bmj.com/site/about/guidelines.xhtml

Depending on the variety of non-pharmacological interventions and studies' populations and on the available data, we plan to investigate differential effects on specific sub-groups (e.g., by age, sex, BP levels, co-morbidities, health-related quality of life), and from different types of interventions (e.g., by duration, setting, mode of delivery) using meta-regression. Where this quantitative approach is not possible, we will discuss the sub-group effects narratively.

Assessment of certainty of evidence

Where relevant, we will assess the level of certainty of the evidence found in the included studies using the GRADE framework.[21]

PATIENT AND PUBLIC INVOLVEMENT

Patients and the public were not directly involved in designing this systematic review protocol. The systematic review is connected to a larger programme of work called REVERSE (Risk Education InterVEntion for Raised blood preSsurE). REVERSE aims to investigate the feasibility of blood pressure self-monitoring for people with prehypertension, which was co-produced with members of the public with prehypertension and hypertension. The results of both the systematic review of non-pharmacological interventions and feasibility study of self-monitoring will help with co-producing future lifestyle interventions, with the aim of preventing hypertension and associated conditions, which will be tested in future effectiveness studies.

ETHICS AND DISSEMINATION

Ethical approval is not needed for this systematic review. The review is registered with Prospero (CRD420232433047), and any important protocol amendments will be updated on the record. It is intended to publish the completed review in a peer-reviewed journal. Results will also be disseminated via the REVERSE study website and will be shared with the REVERSE study sites and participants.

TWITTER

@ReverseStudy

WEBSITE

www.reversestudy.co.uk

AUTHORS CONTRIBUTIONS

EB was responsible for the conception, design and writing of the manuscript. LH, JS, EB, RG, CW, VB, NW, PR and AC contributed to the conception and design. EB, LH, VB and NI contributed to the writing of the manuscript. CH designed the search strategy and performed the preliminary database searches. All authors read and approved the final manuscript. EB is the guarantor of the review.

For peer review only - http://bmjopen.bmj.com/site/about/guidelines.xhtml

FUNDING STATEMENT

EB holds a NIHR RfPB grant (NIHR201028). This work is related to this funding but is not directly funded by it. The views expressed are those of the author(s) and not necessarily those of the NIHR or the Department of Health and Social Care.

COMPETING INTERESTS STATEMENT

VB, CH, AC, JS and CW are part funded by the National Institute for Health Research Applied Research Collaboration North West Coast (NIHR ARC NWC). The views expressed are those of the authors and not necessarily those of the NHS, the NIHR, or the Department of Health and Social Care.

For peer review only

REFERENCES

[1] Wang Q, Wang Z, Yao W, Wu X, Huang J, Huang L, et al. Anthropometric indices predict the development of hypertension in normotensive and pre- hypertensive middle-aged women in Tianjin, China: A prospective cohort study. *Medical Science Monitor.* 2018;24:1871-9.

[2] Zheng L, Sun Z, Zhang X, Xu C, Li J, Hu D, et al. Predictors of progression from prehypertension to hypertension among rural Chinese adults: Results from Liaoning Province. *European Journal of Cardiovascular Prevention and Rehabilitation*. 2010;17(2):217-22.

[3] Huang Y, Su L, Cai X, Mai W, Wang S, Hu Y, et al. Association of all-cause and cardiovascular mortality with prehypertension: A meta-analysis. *American Heart Journal*. 2014;167(2):160-8.e1.

[4] Guo X, Yingxian S. Quantitative association between prehypertension and different types of cardiovascular outcomes and diabetes: A systematic review and meta-analysis of prospective studies worldwide. *Heart*. 2013;3:A115.

[5] Li Y, Xia P, Xu L, Wang Y, Chen L. A Meta-analysis on prehypertension and chronic kidney disease. *PLoS ONE*. 2016;11(6):e0156575.

[6] Kanno A, Kikuya M, Ohkubo T, Hashimoto T, Satoh M, Hirose T, et al. Pre-hypertension as a significant predictor of chronic kidney disease in a general population: The Ohasama Study. *Nephrology Dialysis Transplantation*. 2012;27(8):3218-23.

[7] Huang Y, Cai X, Liu C, Zhu D, Hua J, Hu Y, et al. Prehypertension and the risk of coronary heart disease in Asian and western populations: A meta-analysis. *Journal of the American Heart Association*. 2015;4(2):e001519.

[8] Lee M, Saver JL, Chang B, Chang KH, Lin K, Ovbiagele B. Baseline pre-hypertension and risk of incident stroke: A meta-analysis. *Stroke*. 2011;42(3):e49-e50.

[9] Winegarden CR. From “prehypertension” to hypertension? Additional evidence. *Annals of Epidemiology*. 2005;15(9):720-5.

[10] Joffres M, Falaschetti E, Gillespie C, Robitaille C, Loustalot F, Poulter N, et al. Hypertension prevalence, awareness, treatment and control in national surveys from England, the USA and Canada, and correlation with stroke and ischaemic heart disease mortality: a cross-sectional study. *BMJ Open*. 2013;3:e003423.

[11] Collier SR, Landram MJ. Treatment of prehypertension: lifestyle and/or medication. *Vascular Health and Risk Management.* 2012;8:613-19.

[12] National Health Service. *The NHS Long Term Plan*. Available at: https://www.longtermplan.nhs.uk/publication/nhs-long-term-plan/ [Accessed 19th February 2019].

[13] Whelton PK, Carey RM, Aronow WS, Casey DE Jr, Collins KJ, Dennison Himmelfarb C, et al. 2017 ACC/AHA/AAPA/ABC/ACPM/AGS/APhA/ASH/ASPC/NMA/PCNA guideline for the prevention, detection, evaluation, and management of high blood pressure in adults: executive summary: a report of the American College of Cardiology/American Heart Association Task Force on Clinical Practice Guidelines. *Hypertension*. 2018;71:e13-e115.

[14] Duangpunmat U, Kalampakorn S, Pichayapinyo P. An effect of walking exercise applying the theory of planned behavior in people at risk of hypertension. J Med Assoc Thai. . 2013, 96 Suppl 5

[15] Azadpour N, Tartibian B, Kosar SN. Effects of aerobic exercise training on ACE and ADRB2 gene expression, plasma angiotensin II level, and flow-mediated dilation: a study on obese postmenopausal women with prehypertension. Menopause. . 2017;24(3):269-277.

[16] Moore LL, Visioni AJ, Qureshi MM, Bradlee ML, Ellison RC, D-Agostino R. Weight loss in overweight adults and the long-term risk of hypertension: The Framingham Study. *Archives of Internal Medicine*. 2005;165:1298–303.

For peer review only

[17] Dorough AE, Winett RA, Anderson ES. Davy BM, Martin EC, Hedrick V. DASH to wellness: emphasizing self-regulation through e-health in adults with prehypertension. Health psychology. 2014; 33 (3): 249-54.

[18] Rahimi ARO, Mhmoodpoor A, Sanaie S. The effect of high-calcium and high-potassium diet on grade-I hypertension and high normal blood pressure. Pakistan Journal of Medical Sciences. 2007; 23 (4); 589-592.

[19] Wang SZ, Li S, Xu XY, Lin GP, Shao L, Zhao Y, Wang TH. Effect of slow abdominal breathing combined with biofeedback on blood pressure and heart rate variability in prehypertension. Journal of alternative and complementary medicine. 2010. 16 (10); 1039-45.

[20] Abdullah A, Bakri S, Rasyid H, Aman M, Kasim H, Harjianti T, dan Tendean P, Seweng A. Effects of deep breath on blood pressure in normo-prehypertensive subjects. European Journal of Molecular and Clinical Medicine. 2021; 8 (2): 1636-1642.

[21] Singh, V. P.; Kh; elwal, B.; Effectiveness Of Yoga and Lifestyle Modification On Prehypertensive Subjects-A Randomised Controlled Trial. NeuroQuantology. 2022. 20 (17); 1323-1337.

[22] Ankolekar VH, Reddy GG, Sanju CSV, Mamatha H. Role of yoga intervention on quality of life and prehypertension. *Indian J Tradit Know.* 2019: 18:351–5.

[23] He, Jiang[a,b]; Streiffer, Richard H[c]; Muntner, Paul[a,b]; Krousel-Wood, Marie A[c]; Whelton, Paul K[a,b]. Effect of dietary fiber intake on blood pressure: a randomized, double-blind, placebo-controlled trial. Journal of Hypertension 22(1):p 73-80, January 2004.

[24] Svetkey LP, Erlinger TP, Vollmer WM, Feldstein A, Cooper LS, Appel LJ, Ard JD, Elmer PJ, Harsha D, Stevens VJ. Effect of lifestyle modifications on blood pressure by race, sex, hypertension status, and age, Journal of human hypertension. 2005: 19 (1); 21-31.

[25] Chiu YH, Wu SC, Tseng CD, Yen MF, Chen TH. Progression of pre-hypertension, stage 1 and 2 hypertension (JNC 7): a population-based study in Keelung, Taiwan (Keelung Community-based Integrated Screening No. 9). Journal of Hypertension. 2006;24(5):821-8.

[26] Padilla J, Wallace JP, Park S. Accumulation of physical activity reduces blood pressure in pre- and hypertension. Medicine and Science in Sports and Exercise. 2005;37:1264–75.

[27] Mehrnoosh E, Atefeh A, Sahar V, Majid G, Iqbal DR, Yamuna S. Effect of Different Types of Aerobic Exercise on Individuals With and Without Hypertension: An Updated Systematic Review. Current problems in cardiology. 2023: 48 (3); 101034.

[28] Shao T, Liang L, Zhou C, Tang Y, Gao W, Tu Y, Yin Y, Malone DC, Tang W. Short-term efficacy of non-pharmacological interventions for global population with elevated blood pressure: A network meta-analysis Frontiers in public health. 2022. 10; 1051581.

[29] Fu J, Liu Y, Zhang L, Zhou L, Li D, Quan H, et al. Nonpharmacologic Interventions for Reducing Blood Pressure in Adults With Prehypertension to Established Hypertension. *Journal of the American Heart Association.* 2020;9(19):e016804.

[30] Lefebvre C, Glanville J, Briscoe S, Featherstone R, Littlewood A, Marshall C, et al. Chapter 4: Searching for and selecting studies. In: Higgins JPT, Thomas J, Chandler J, Cumpston M, Li T, Page MJ, Welch VA (editors). *Cochrane Handbook for Systematic Reviews of Interventions* version 6.3 (updated February 2022). Cochrane, 2022. Available: www.training.cochrane.org/handbook.

[31] University of York Centre for Reviews and Dissemination. How are studies identified for NHS EED. 2009. Available: https://www.crd.york.ac.uk/crdweb/searchstrategies.asp [Accessed 15 May 2023].

[32] Evers S, Goossens M, De Vet H, Van Tulder M, Ament A. Criteria list for assessment of methodological quality of economic evaluations: Consensus on Health Economic Criteria. *International Journal of Technology Assessment in Health Care*. 2005;21(2):240-245.

For peer review only - http://bmjopen.bmj.com/site/about/guidelines.xhtml

[33] Schünemann HJ, Higgins JPT, Vist GE, Glasziou P, Akl EA, Skoetz N, Guyatt GH; on behalf of the Cochrane GRADEing Methods Group and the Cochrane Statistical Methods Group. Cochrane Handbook for Systematic Reviews of Interventions version 6.3, Section 14.3. 2022. Available: https://training.cochrane.org/handbook/current/chapter-14#section-14-3.

For peer review only

For peer review only - http://bmjopen.bmj.com/site/about/guidelines.xhtml

Non-Pharmacological intervention for PHT systematic review

Medline Search Strategy

Search conducted May 26th 2023

Search Number	Query	Hits
1	Prehypertension/	1144
2	prehypertensi*.ti,ab,kw,kf.	2946
3	pre-hypertensi*.ti,ab,kw,kf.	925
4	(pre-htn or prehtn).tw.	113
5	((stage 1 or stage one) adj hypertensi*).tw.	666
6	(elevated blood pressure or elevated bp).ti.	866
7	high-normal blood pressure.tw.	409
8	raised blood pressure.ti.	83
9	borderline hypertensi*.tw.	1403
10	early hypertensi*.tw.	365
11	raised systolic blood pressure.tw.	75
12	raised diastolic blood pressure.tw.	16
13	("at risk" adj1 hypertensi*).tw.	43
14	((("120" adj2 "129") or ("130" adj2 "139") or ("120" adj2 "139") or ("80" adj2 "89")) and (blood pressure or bp)).tw.	964
15	or/1-14	7975
16	exp randomized controlled trial/	594769
17	controlled clinical trial.pt.	95314
18	randomi#ed.ab.	721331
19	placebo.ab.	238387
20	randomly.ab.	408822
21	trial.ab.	649200
22	groups.ab.	2520111
23	or/16-22	3638767
24	exp animals/ not humans.sh.	5123796
25	23 not 24	3135655
26	15 and 25	2372

For peer review only

Reporting checklist for protocol of a systematic review and meta analysis.

Based on the PRISMA-P guidelines.

Instructions to authors

Complete this checklist by entering the page numbers from your manuscript where readers will find each of the items listed below.

Your article may not currently address all the items on the checklist. Please modify your text to include the missing information. If you are certain that an item does not apply, please write "n/a" and provide a short explanation.

Upload your completed checklist as an extra file when you submit to a journal.

In your methods section, say that you used the PRISMA-Preporting guidelines, and cite them as:

Moher D, Shamseer L, Clarke M, Ghersi D, Liberati A, Petticrew M, Shekelle P, Stewart LA. Preferred Reporting Items for Systematic Review and Meta-Analysis Protocols (PRISMA-P) 2015 statement. Syst Rev. 2015;4(1):1.

		Reporting Item	Page Number
**Title**			
Identification	#1a	Identify the report as a protocol of a systematic review	1
Update	#1b	If the protocol is for an update of a previous systematic review, identify as such	n/a
**Registration**			
	#2	If registered, provide the name of the registry (such as 7PROSPERO) and registration number	2 & 7
**Authors**			
Contact	#3a	Provide name, institutional affiliation, e-mail address of all protocol authors; provide physical mailing address of corresponding author	1
Contribution	#3b	Describe contributions of protocol authors and identify the guarantor of the review	7

For peer review only

**Amendments**			
	#4	If the protocol represents an amendment of a previously completed or published protocol, identify as such and list changes; otherwise, state plan for documenting important protocol amendments	7
**Support**			
Sources	#5a	Indicate sources of financial or other support for the review	8
Sponsor	#5b	Provide name for the review funder and / or sponsor	8
Role of sponsor or funder	#5c	Describe roles of funder(s), sponsor(s), and / or institution(s), if any, in developing the protocol	8
**Introduction**			
Rationale	#6	Describe the rationale for the review in the context of what is already known	3
Objectives	#7	Provide an explicit statement of the question(s) the review will address with reference to participants, interventions, comparators, and outcomes (PICO)	3
**Methods**			
Eligibility criteria	#8	Specify the study characteristics (such as PICO, study design, setting, time frame) and report characteristics (such as years considered, language, publication status) to be used as criteria for eligibility for the review	4-5
Information sources	#9	Describe all intended information sources (such as electronic databases, contact with study authors, trial registers or other grey literature sources) with planned dates of coverage	4
Search strategy	#10	Present draft of search strategy to be used for at least one electronic database, including planned limits, such that it could be repeated	Appendix 1
Study records - data management	#11a	Describe the mechanism(s) that will be used to manage records and data throughout the review	4
Study records - selection process	#11b	State the process that will be used for selecting studies (such as two independent reviewers) through each phase of the review (that is, screening, eligibility and inclusion in meta-analysis)	4
Study records - data	#11c	Describe planned method of extracting data from reports (such as	5-6

For peer review only

collection process		piloting forms, done independently, in duplicate), any processes for obtaining and confirming data from investigators	
Data items	#12	List and define all variables for which data will be sought (such as PICO items, funding sources), any pre-planned data assumptions and simplifications	6
Outcomes and prioritization	#13	List and define all outcomes for which data will be sought, including prioritization of main and additional outcomes, with rationale	6
Risk of bias in individual studies	#14	Describe anticipated methods for assessing risk of bias of individual studies, including whether this will be done at the outcome or study level, or both; state how this information will be used in data synthesis	6
Data synthesis	#15a	Describe criteria under which study data will be quantitatively synthesised	6-7
Data synthesis	#15b	If data are appropriate for quantitative synthesis, describe planned summary measures, methods of handling data and methods of combining data from studies, including any planned exploration of consistency (such as I2, Kendall's τ)	6-7
Data synthesis	#15c	Describe any proposed additional analyses (such as sensitivity or subgroup analyses, meta-regression)	6-7
Data synthesis	#15d	If quantitative synthesis is not appropriate, describe the type of summary planned	n/a
Meta-bias(es)	#16	Specify any planned assessment of meta-bias(es) (such as publication bias across studies, selective reporting within studies)	6
Confidence in cumulative evidence	#17	Describe how the strength of the body of evidence will be assessed (such as GRADE)	6

None The PRISMA-P elaboration and explanation paper is distributed under the terms of the Creative Commons Attribution License CC-BY. This checklist can be completed online using https://www.goodreports.org/, a tool made by the EQUATOR Network in collaboration with Penelope.ai

For peer review only
